# Leonard Newsom Morris

**Published:** 1945

**Authors:** 


### LEONARD NEWSOM MORRIS, M.B., Ch.B., F.R.C.S., M.R.C.S., L.R.C.P.

THE death occurred on 28th December of Mr. Leonard Newsom Morris. He studied at Bristol Medical School and became F.R.C.S. (Edin.), M.R.C.S., L.R.C.P. (London), and M.B., Ch.B. (Bristol), and was for a time house surgeon and house physician at Bristol General Hospital, and later became one of the senior (in-patient) surgeons at Bristol Children's Hospital.

He died within a few hours of the death of his wife, and leaves no children.

